# The weight of choices: Prioritizing lifestyle over GLP-1 receptor agonist therapy in managing MASLD

**DOI:** 10.1016/j.jhepr.2025.101401

**Published:** 2025-03-26

**Authors:** Jörn M. Schattenberg, Jacob George

**Affiliations:** 1 Department of Medicine II, University Medical Center Homburg, Homburg and Saarland University, Saarbrücken, Germany; 2 Storr Liver Centre, The Westmead Institute of Medical Research, Westmead Hospital and University of Sydney, Sydney, Australia

As metabolic dysfunction-associated steatotic liver disease (MASLD) has grown to become the most prevalent liver disease in the general^1^ and at-risk populations,^2^ with high socioeconomic costs,^3^ the race for effective treatments and cure is on. Next to the somatic and health system burden, impaired quality of life also needs to be highlighted.^4^^,^^5^ Achieving and maintaining weight loss are the foundational recommendations in all guidelines. While most physicians will highlight the benefits of weight loss^6^ and most people affected by MASLD are cognizant of the beneficial effects of weight loss in metabolic disease, recommendations “on the fly” as frequently given during short outpatient clinic visits are *de facto* without any lasting benefit. On the contrary, this approach can lead to self-doubt and frustration, negatively impacting the physician-patient relationship. Therefore, effective weight-loss and weight maintenance strategies are urgently required. An approach that factors in disease stage assessed non-invasively, personal preferences, clearly communicated treatment goals and support have a higher chance of success (Fig. 1).

**Fig. 1:**
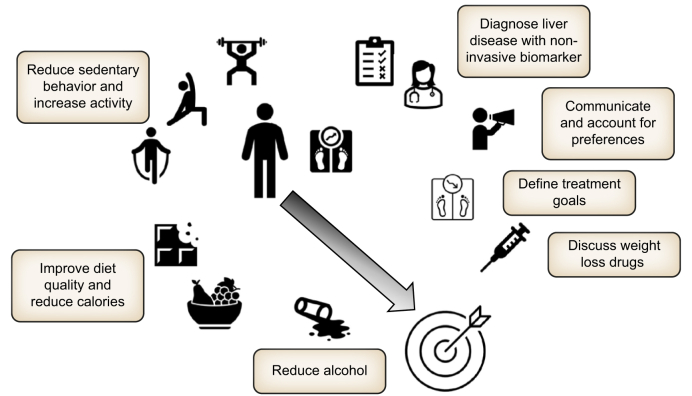
Individualized treatment goals in MASLD include lifestyle, diet and pharmacological treatment options to support weight loss and improve the hepatic phenotype. Diet and behavior, personal preference, stage of liver disease, costs and safety are determinants. MASLD, metabolic dysfunction-associated steatotic liver disease.

The evidence for lifestyle modifications and weight loss drugs on steatohepatitis and fibrosis is strong, as is the evidence at a more holistic level for diabetes prevention and treatment, and for cardiovascular risk reduction. The study of Moolla and colleagues^7^ conducted in the UK compared the effectiveness of a 12-week lifestyle intervention *vs*. liraglutide (1.8 mg weekly) in individuals with phenotypic MASLD. Matched weight loss was achieved from both a 500 kcal energy restriction and GLP-1RA treatment, with similar improvements in body composition and surrogates of liver disease activity. However, it should be noted that those receiving the GLP1-RA also had significant reductions in lean body mass. These results underscore the effectiveness of lifestyle changes – such as diet modification and increased physical activity – in improving liver health and achieving the effect size of the first-generation GLP-1RAs.

Both interventions also carry significant challenges for patients and healthcare providers and safety is a key issue in the management of metabolic diseases. Even with this short treatment in a highly controlled experimental setting, the withdrawal rate was 13% for the lifestyle intervention and 20% for the GLP-1RA group. This emphasizes the need for frequent consultations and feedback to maintain patients on treatment. The *a priori* definition of achievable goals that are mutually defined and can require adaptations will support treatment success. In the indication for type 2 diabetes, discontinuation rates for GLP-1 analogues were up to 45% at 12 months.^8^ The same is true for hypocaloric interventions, as individual factors and diet characteristics contribute to high discontinuation rates.^9^ For this reason, a diet program that aligns with personal preferences and could include other strategies, *e.g.* gluten reduction or intermittent fasting as preferred options needs to be considered.^10^^,^^11^

Another important aspect to consider is the potency of the pharmacological intervention. The older GLP-1RA liraglutide showed a moderate effect on weight loss in the range of 4-5% at 12 weeks. With the most recent dual and triple GLP-1RAs, these effects can exceed 10% at 12 weeks and, depending on the combination, promising liver effects have been observed.^12^ The liver phenotypes were captured well in the current study, including cT1, MRI and transient elastography, and multiple metabolic and experimental biomarkers. The short-term improvement of the biomarkers is reasonably likely to transfer into long-term benefit and outcome with regards to liver and overall health, as has already been reported for cardiovascular risk reduction in the SELECT trial.^13^ The observed effects on blood pressure are a strong rationale to promote any of the two interventions. The improvement in diastolic blood pressure with lifestyle interventions but not liraglutide could be recapitulated by the next-generation dual agonist GLP-1RAs, which have also been shown to reduce blood pressure.^14^

An interesting aspect of this study is the exploration of legacy effects of either intervention over 12-weeks, with a modest weight increase seen in the GLP-1RA group, but additional weight loss occurring after lifestyle intervention. This finding argues for a more pronounced metabolic change with lifestyle intervention, as indicated by metabolic and lipogenic changes as well as experimental markers. Importantly, lifestyle interventions empower individuals to take charge of their health, fostering a sense of agency that is often lacking with pharmacological treatments. The withdrawal of GLP-1RAs after 12 weeks raises concerns regarding their impact on the body’s metabolic landscape. Elevated levels of circulating MMP-10, IL10RB, FGF-23, and Flt3L, alongside dysregulated adipose tissue gene expression, indicate a potential rebound effect that could predispose individuals to weight regain. This phenomenon highlights a critical issue in the use of pharmacological interventions: the risk of dependency and the potential for adverse metabolic consequences upon cessation. More importantly, the sum of data suggests that neither treatment on its own is likely to be optimal, rather, even in the context of pharmacotherapy, emphasis should be placed on lifestyle education, internalizing change and ongoing support. The focus of lifestyle intervention should emphasize optimal weight maintenance, diet quality, physical activity (aerobic, resistance and balance) and reduced sedentary behavior (Fig. 1).

While the follow-up period was short, it demonstrates the underlying dilemma of pharmacotherapy – the lack of sustainability of the benefits if the agent is stopped. The current study therefore underlines the strong case for non-pharmacological approaches. Given the findings of this trial, it is imperative that we advocate for lifestyle modifications as the first-line approach in managing MASLD. Non-pharmacological therapies – adapted and maintained – not only offer a sustainable solution to weight management but also promote a holistic view of health that encompasses physical, emotional, and social well-being. By prioritizing lifestyle changes, we empower individuals to make lasting adjustments to their daily habits, fostering resilience against the chronic nature of MASLD and of overall metabolic health. Moreover, the cost-effectiveness of lifestyle interventions cannot be overlooked. Nonetheless, pharmacological therapies are urgently needed for the large group of individuals with MASLD who are unable to achieve and maintain the treatment goals of non-pharmacologic therapy. A balanced approach is required (Fig. 1). While the trial highlights both the efficacy and safety of lifestyle modifications, their combination with pharmacotherapy will likely be required for most patients to achieve systemic metabolic and liver benefits.

## Financial support

No financial support.

## Authors’ contributions

Both authors conceptualized and wrote the comment

## Conflict of interest

JMS declares consultant honorary from Akero, Alentis, Alexion, Altimmune, Astra Zeneca, 89Bio, Bionorica, Boehringer Ingelheim, Boston Pharmaceuticals, Gilead Sciences, GSK, HistoIndex, Ipsen, Inventiva Pharma, Madrigal Pharmaceuticals, PRO.MED.CS Praha a.s., Kríya Therapeutics, Eli Lilly, MSD Sharp & Dohme GmbH, Novartis, Novo Nordisk, Pfizer, Roche, Sanofi, Siemens Healthineers; speaker honorarium from AbbVie, Boehringer Ingelheim, Gilead Sciences, Ipsen, Lilly, Novo Nordisk, Madrigal Pharmaceuticals, Stockholder options: Hepta Bio.
